# Supplementary material for: Microtubule-associated proteins MAP7 and MAP7D1 promote DNA double-strand break repair in the G1 cell cycle phase
Source: iScience. 2023 Feb 1;26(3):106107. doi: 10.1016/j.isci.2023.106107 (PMC9958362; doi:10.1016/j.isci.2023.106107)
Supplement: Document S1. Figures S1 and S2 [file mmc1.pdf]

iScience, Volume *26*

Supplemental information

Microtubule-associated proteins MAP7 and MAP7D1 promote DNA double-strand break repair in the G1 cell cycle phase

Arlinda Dullovi, Meryem Ozgencil, Vinothini Rajvee, Wai Yiu Tse, Pedro R. Cutillas, Sarah A. Martin, and Zuzana Hořejší

Figure S1: MAP7 interacts with N-terminal parts of DDR proteins MLH1 and XPC. MAP7D1 interacts with BRCA1 and RAD50. Related to Figure 2.

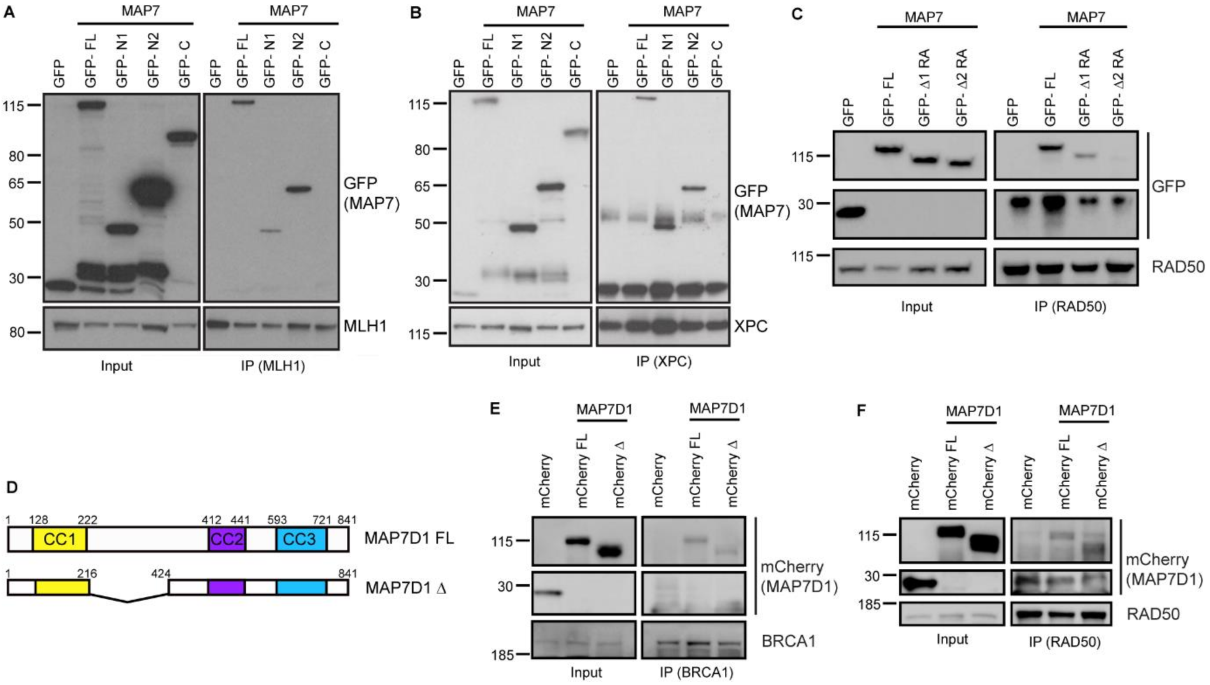

(A) Domain mapping by immuno-precipitation of full-length GFP-MAP7 (GFP-FL) and MAP7 truncation constructs by endogenous MLH1 from HEK293 extract overexpressing GFP-MAP7 constructs. (B) Domain mapping by immuno-precipitation of full-length GFP-MAP7 (GFP-FL) and GFP-MAP7 truncation constructs by endogenous XPC from HEK293 extract overexpressing GFP-MAP7 constructs. (C) Immuno-precipitation of GFP-MAP7 constructs mutated on Arg144/145 to Ala with deletion between aa151-270 (GFP-ΔN1 RA) and 151-300 (GFP-ΔN2 RA) with endogenous RAD50. (D) Schematic representation of full-length MAP7D1 and MAP7D1 deletion construct used in immunoprecipitations. (E) Immuno-precipitation of mCherry-tagged MAP7D1 wt and deletion mutation by endogenous BRCA1 from HEK293T extract overexpressing mCherry constructs. (F) Immuno-precipitation of mCherry-tagged MAP7D1 wt and deletion mutation by endogenous RAD50 from HEK293T extract overexpressing mCherry constructs.

Figure S2. Downregulation of MAP7 and MAP7D1 leads to increased p53 S15 phosphorylation in A549 cells. Related to Figure 6.

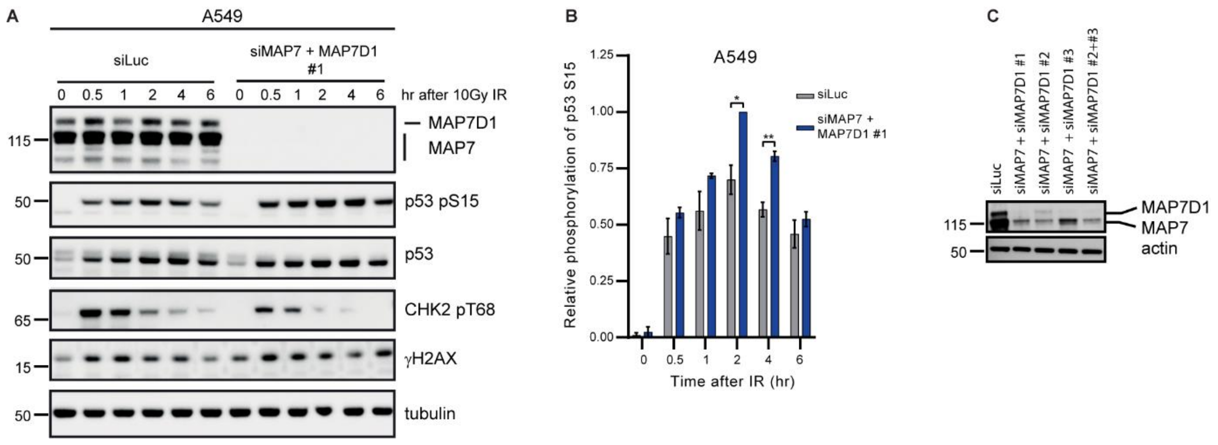

(A) Western blot analysis of DNA damage response after 10Gy of gamma-irradiation in A549 cells treated with siRNA targeting luciferase or MAP7 and MAP7D1 at different timepoints. (B) Quantification of relative phosphorylation of p53 on Ser15 after γ-irradiation in A549 cells treated with siRNA targeting luciferase or MAP7 and MAP7D1. Signal intensity quantification was performed by ImageJ software. The p-Ser15 of p53 signal intensity was normalised against β-actin and expressed as relative phosphorylation of p53 at Ser15. Results are presented as mean ± SEM of values from three independent experiments. Statistical differences were determined from unpaired *t*-test: $p<0.05$, * $p<0.01$, **. (C) Western blot analysis of MAP7 and MAP7D1 siRNA treatment in MCF7 cell line.
